# Optical bistability via an external control field in all-fiber ring cavity

**DOI:** 10.1038/s41598-017-09570-x

**Published:** 2017-08-21

**Authors:** Shili Li, Qiang Ge, Zhiping Wang, Juan Carlos Martín, Benli Yu

**Affiliations:** 1 Key Laboratory of Opto‐Electronic Information Acquisition and Manipulation of Ministry of Education, Anhui University, Hefei, 230601 China; 2 School of Mathematics and Physics, Anhui Polytechnic University, Wuhu, 241000 China; 3 Applied Physics Dept. and I3A, Universidad de Zaragoza, 50009 Zaragoza, Spain

## Abstract

We demonstrate a new scheme for realizing the Optical Bistability (OB) inside an all-fiber ring cavity with an external control field. In the absence of the external control field, the pump power is fixed below the threshold value of laser, and there is no laser in the cavity. However, when the control signal of 1505 nm to 1520 nm is injected into the cavity, laser begins to oscillate and OB appears. We found that the wavelength and power of the control signal can affect the OB behavior dramatically, which can be used to manipulate efficiently the threshold intensity and the hysteresis loop. We also give an explanation of the bistability phenomenon based on numerical simulations, which are agreed very well with our experimental results. Our scheme may provide some new possibilities for technological applications in optical power limiters, switches or memories.

## Introduction

In the past few decades, Optical Bistability (OB) has been the subject of many recent studies because of its potential wide applications in optical transistor, memory element, and all-optical switching^1–8^. Meanwhile, many researchers have been carried out to obtain OB in erbium-doped fiber laser (EDFL)^9–15^. For example, Jung Mi Oh and Donghan Lee demonstrated strong optical bistability in a widely tunable L-band erbium-doped fiber ring laser pumped by a 980-nm laser diode. They found that the bistable region is as much as 150-mW wide, and which can be controlled by the lasing wavelength or the length of erbium-doped fiber^12^. Shao *et al*. investigated OB in a single fiber ring laser. They show that useless pump loss and active spontaneous emission (ASE) can eventually resulted in the bistability phenomenon^13^. Later on, optical bistability in saturable absorber-based single-frequency Brillouin fiber laser has also been experimentally investigated^14^. Quite recently, Juan C. Martín studied a sine-wave-modulated erbium-doped-fiber laser with an external signal coupled into its cavity and analyzed how the time-dependent laser emission changes as a function of the external signal power. Under appropriate working conditions, different bistable behaviors can be found^15^.

In this work, we investigate the OB inside an all-fiber ring cavity with an external control field. Our work is mainly based on the^13, 14^, however, which is drastically different from those works. The major differences are obtained as follows. First and foremost is that we are interested in investigating the Optical bistability inside an all-fiber ring cavity with an external control field when the pump power is fixed below the threshold value of laser. Second, when the control signal was injected into the cavity, laser begins to oscillate and the OB appears. The wavelength and power of the control signal can affect the OB behavior dramatically, which can be used to manipulate efficiently the threshold intensity and the hysteresis loop. Third, we give an explanation of the bistability phenomenon based on numerical simulations, which are agreed very well with our experimental results.

## Experiment

**Figure 1:**
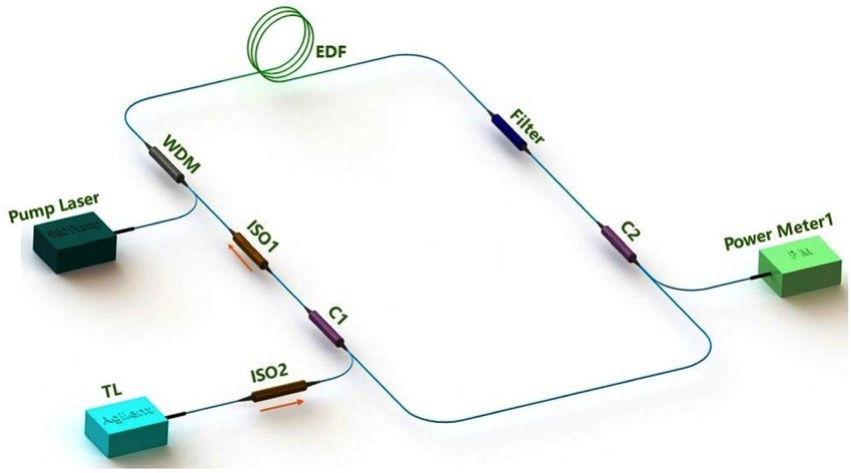
Experimental setup.

The experiment setup is shown in Fig. 1. The optical ring cavity contains six elements: a fiber filter with central wavelength of 1550 nm (3 dB bandwidth is 1.4 nm) which is used to select the wavelength of laser; 30 meters long EDF with 5.20 × 10^24^ m^−3^ erbium-ion concentration; an unidirectional isolator (ISO1), which is used to avoid backward amplified spontaneous emission (ASE); a 80/20 coupler (C1) employed to couple 20% of an external control laser into the ring, while another 80/20 coupler (C2) is used to extract 20% of the laser power in the ring cavity linked to Power Meter1 for detection; a 980 nm/1550 nm Wavelength Division Multiplex (WDM) couples 980 nm pump laser into the ring cavity. The control signal is injected into ring cavity through the Tunable Laser (TL, Agilent 81600B-160), and isolator2 ensures the cavity light does not damage the control laser source.

**Figure 2:**
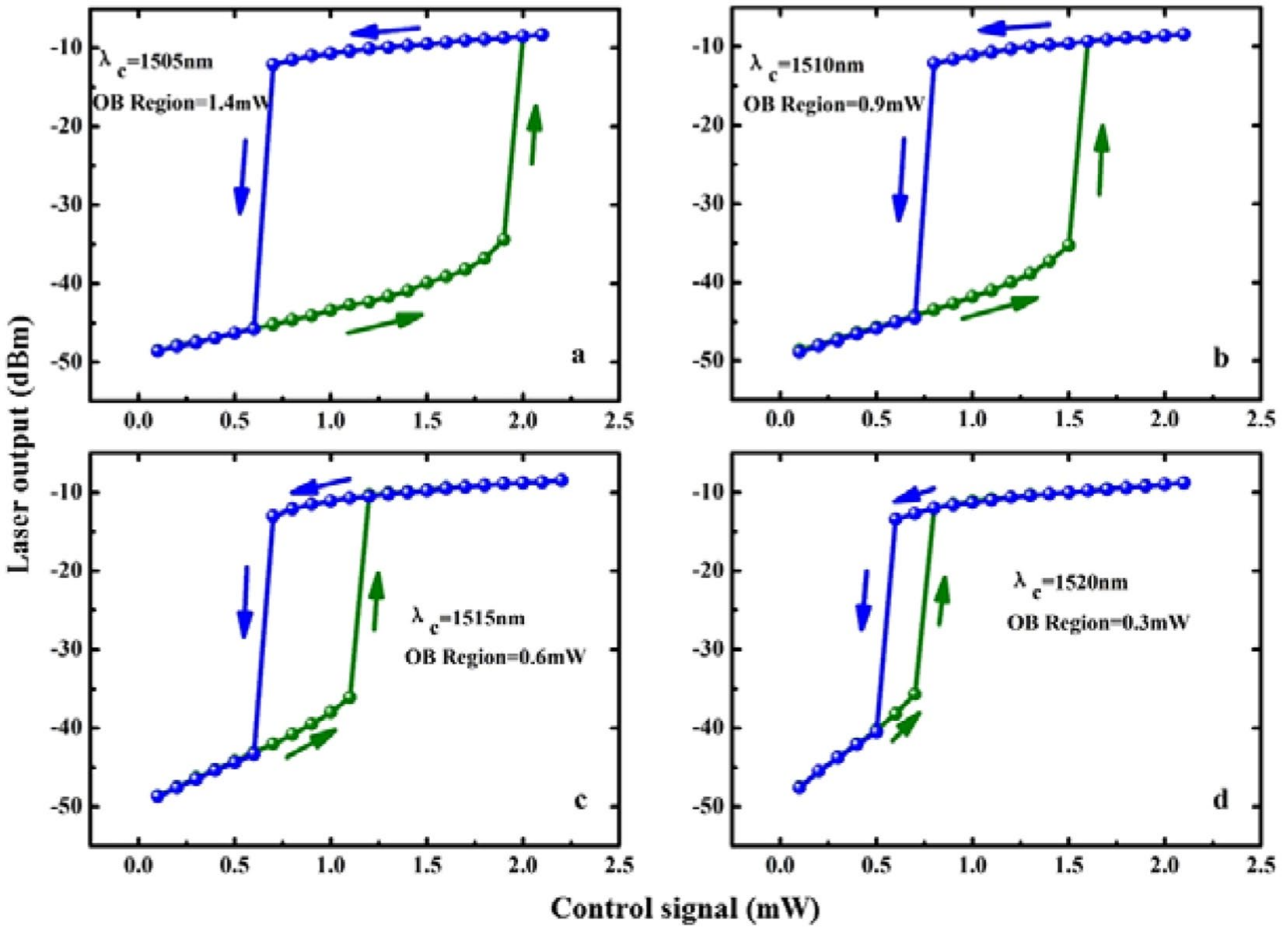
Laser Output power versus control signal for different wavelengths of control signal.

Figure 2 shows the effect of the control signal wavelength on OB when the power of pump laser is 56 mW (threshold of the laser is 59.2 mW). If the wavelength of control signal λ_c_ is fixed at 1505 nm, as shown in Fig. 2(a), the range of OB region is 1.4 mW. When the wavelength of control signal is tuned to λ_c_ = 1510 nm, one can see that the range of OB region decreases from 1.4 mW to 0.9 mW, as shown in Fig. 2(b). As we further increase wavelength of control signal from 1515 nm (see Fig. 2(c)) to 1520 nm (see Fig. 2(d)), we found that both the threshold value and range of OB region decrease. The reason can be qualitatively explained as follows. When the control signal is taken into account, there appears a supplementary pump power that affects the absorption of EDF. So, when the wavelength of control signal is shifted from 1505 nm to 1520 nm, we can see that the threshold value and the width of the hysteresis cycle can be easily changed via modifying the wavelength of the control laser.

**Figure 3:**
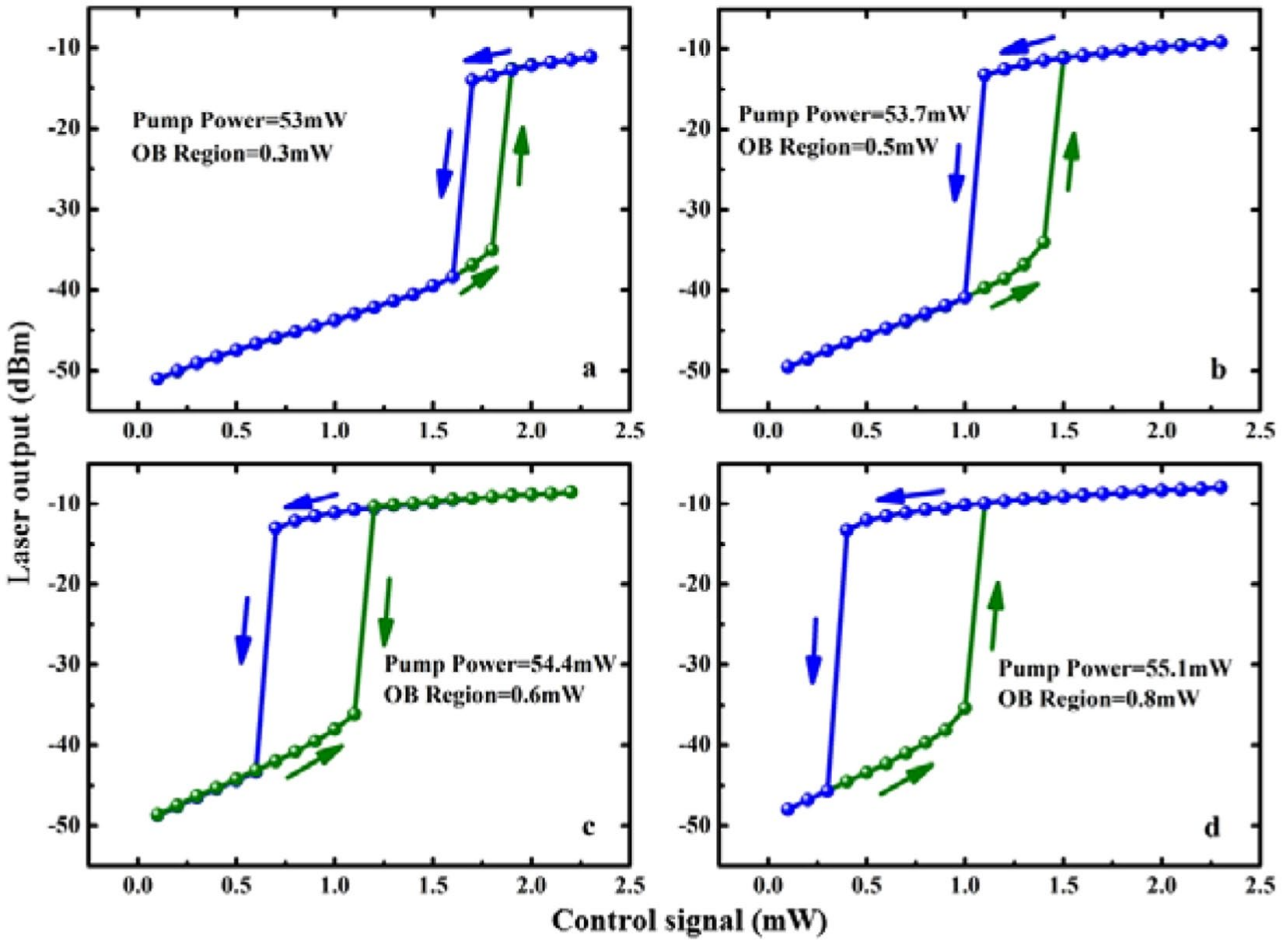
Laser output power versus control signal for different powers of pump laser.

In Fig. 3, we study how the power of pump laser affects on the width of OB region. We plot the laser output power versus control signal power for different values of pump power when the wavelength of the control signal is fixed at λ_c_ = 1515 nm. Clearly, the bistable region depends crucially on the pump power, which might be useful to control the threshold value and the hysteresis cycle width of the OB simply by adjusting the power of pump laser.

## Numerical Results and Discussions

In order to gain deeper insight into the above phenomena, we also give the numerical simulations in this Section. Next, we calculated series of laser responses as a function of the control signal power, first in ascending order and then in descending order (P_c1_, P_c2_, …P_c,n-1_, P_c,n_, P_c,n-1_,… P_c2_, P_c1_). For carrying out the simulations as close as possible to the experimental conditions, every laser response is obtained as the final result of a transient process that is fully calculated. This final result is taken as the initial condition for the calculation of the next transient (in other words, for the calculation of the next point in the series). Transient calculations are carried out by means of the model described in ref. 16. Absorption and emission coefficients have been taken according to the manufacturer’s specifications (Table 1). The transmission factor of the ring passive part (transmission of all the elements except the active fiber plus transmission of the active-passive fiber splices) has been estimated in 0.11 and the lifetime of the ^4^I_13/2_ level of the Er^3+^ ion is 10.5 ms. For the calculations, we also need the radius of the doped transversal area in the active fiber. As the manufacturer does not provide this information, we have tried several values. Here we present the results obtained with a radius of 3.75 μm. The other parameters in the numerical simulations have been specified in the previous experiment section.

**Table 1:** Values of the absorption and emission coefficients at the different control signal wavelengths considered, according to the manufacturer’s specifications.

Wavelength (nm)	Absorption coefficient (dBm)	Emission coefficient (dBm)
1505	2.6	1.3
1510	2.8	1.6
1515	3.0	2.0
1520	3.4	2.5

**Figure 4:**
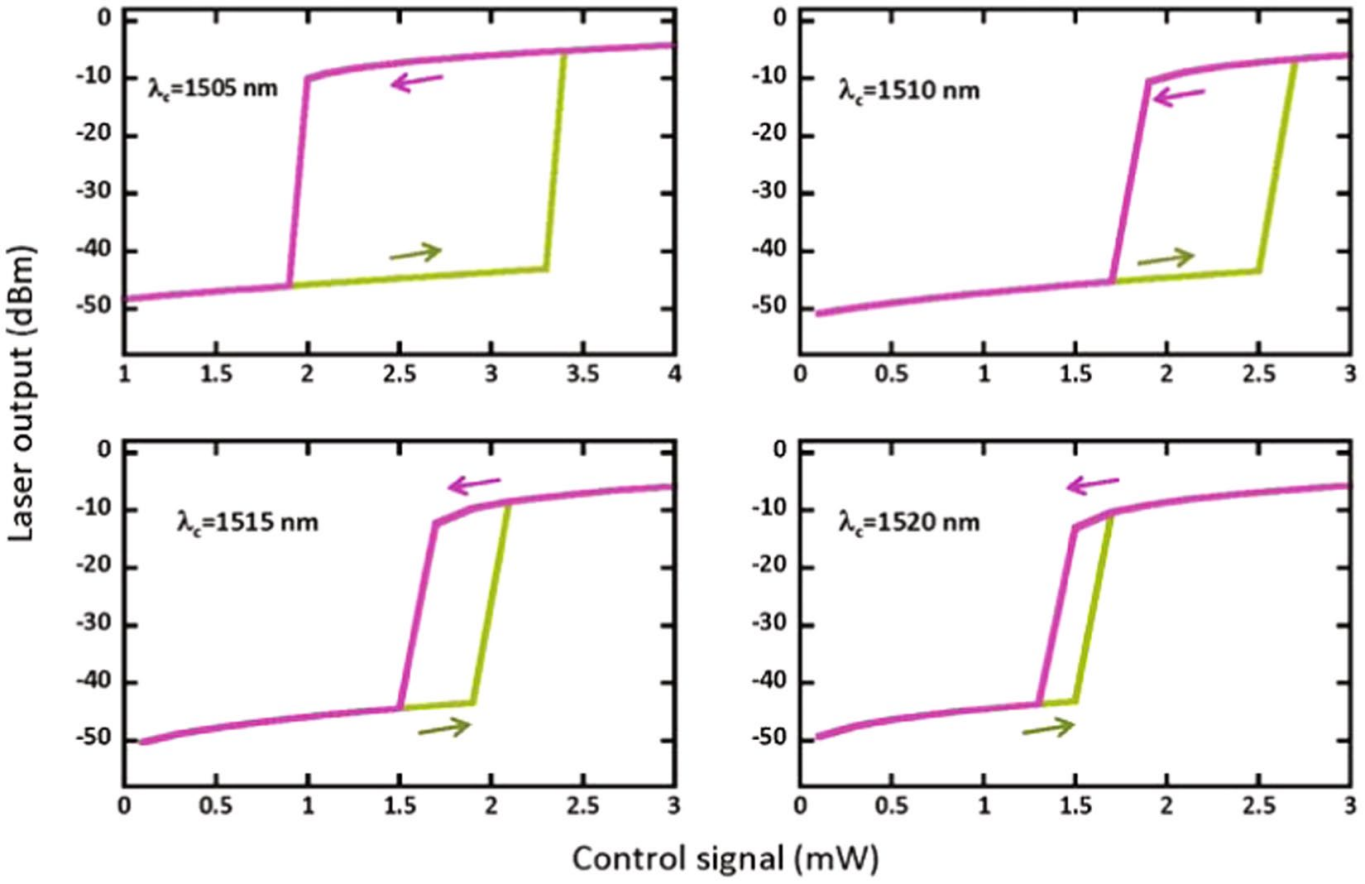
Laser output power versus the control signal for different wavelengths of control signal.

We choose the threshold of pump power is 65 mW (no control signal is injected). So, in order to reproduce the experiment shown in Fig. 2, we consider a case that the pump power slightly lower than the threshold: we tune pump power to 62 mW. From Fig. 4, we can see the results are agreed very well with experimental results in Fig. 2.

**Figure 5:**
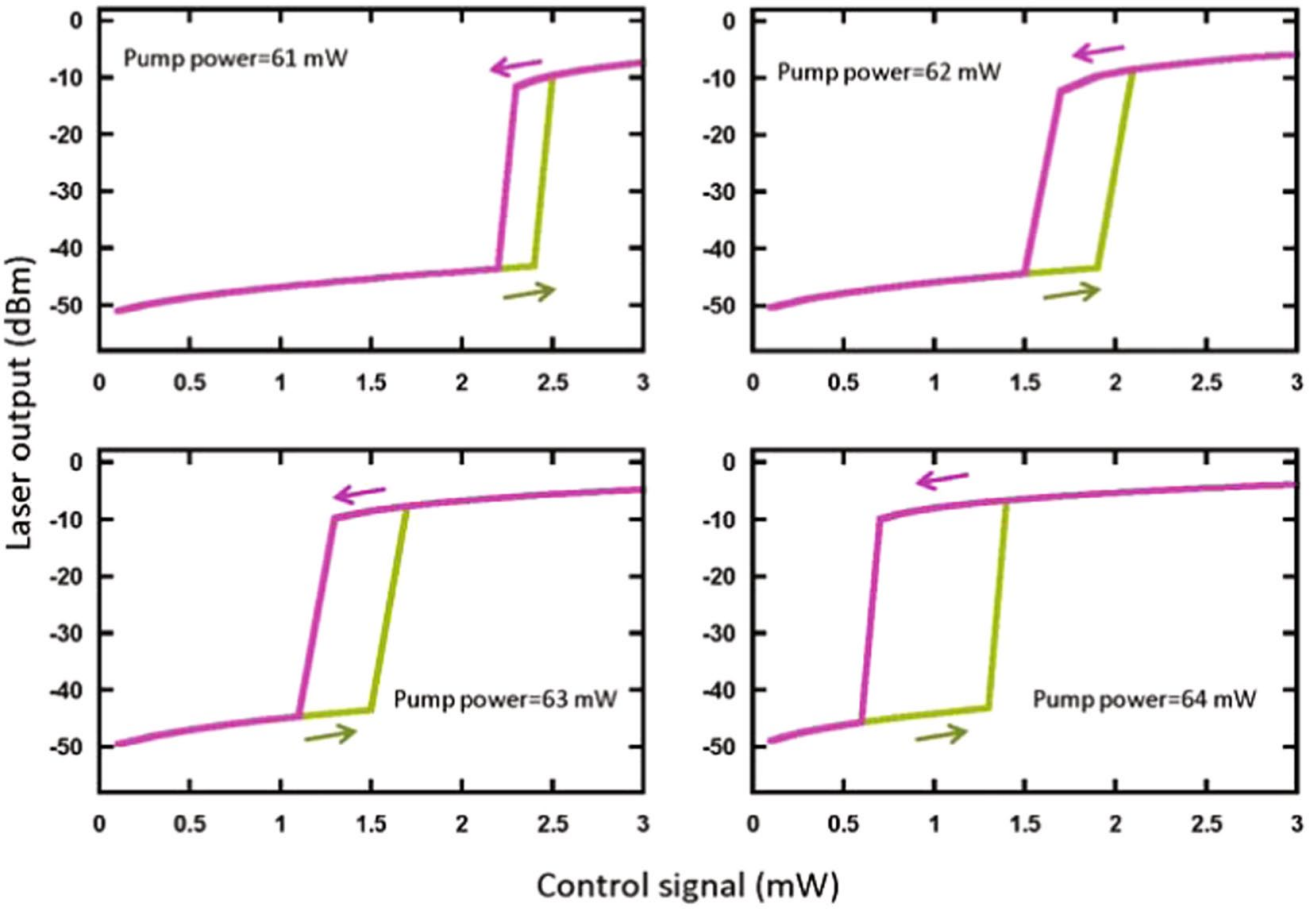
Laser output power versus control signal for different powers of pump laser.

The effect of the pump power on the width of the bistable region has also been numerically simulated in Fig. 5. As shown in Fig. 5, one can find the numerical results are the same as in the experimental results in Fig. 3 (the wavelength of the control signal has been fixed at 1515 nm).

The numerical results provide a clear explanation of the OB observed. Each laser response in CW regime takes place after a transient stage. When all the laser inputs (pump and control power) are stable along time, the laser adopts a certain population distribution and there is a balance between emission and absorption at every point along the fiber. If the power of control signal is changed, the balance is suddenly altered and the population distribution experiences a transient stage until the system reaches a new balance. It is not surprising that the final state of the transient depends on its initial conditions, provided that the EDFL is a strongly nonlinear system, which is clear according to the model explained in ref. 16.

## Conclusions

To sum up, we have investigated Optical Bistability (OB) inside an all-fiber ring cavity with an external control field. The numerical results are also obtained, which are agreed very well with our experimental results. Therefore, it is clear that the model employed accounts for the OB observed. As a qualitative explanation, it may be pointed out that the laser CW response is always the end of a transient process. In a strongly nonlinear system such as an EDFL, it is natural that the end of a transient depends on its initial conditions, determined by the previous CW laser state. Our scheme may provide some new possibilities for technological applications in optical power limiters, switches or memories.
